# Histological Characteristics, Cell Wall Hydrolytic Enzymes Activity and Candidate Genes Expression Associated with Seed Shattering of *Elymus sibiricus* Accessions

**DOI:** 10.3389/fpls.2017.00606

**Published:** 2017-04-19

**Authors:** Xuhong Zhao, Wengang Xie, Junchao Zhang, Zongyu Zhang, Yanrong Wang

**Affiliations:** State Key Laboratory of Grassland Agro-ecosystems, College of Pastoral Agriculture Science and Technology, Lanzhou UniversityLanzhou, China

**Keywords:** *Elymus sibiricus*, seed shattering, histological characteristics, hydrolytic enzymes activity, candidate genes expression, mechanism

## Abstract

*Elymus sibiricus* (siberian wildrye) is a perennial, cool-season, self-pollinating, and allotetraploid grass. As an economically important species, it has been widely grown and used for pasture and hay in northern China. Because of serious seed shattering (SS), however, *E. sibiricus* is difficult to grow for commercial seed production. To better understand the underlying mechanism of SS, we investigated the differences in SS of cultivars and wild accessions in relation to morphological and genetic diversity, histological characteristics, lignin staining, cell wall hydrolytic enzymes activity and candidate genes expressions. We found high level of morphological and genetic diversity among *E. sibiricus* accessions. In general, cultivars had higher average pedicel breaking tensile strength (BTS) value than wild accessions, of which PI655199 had the highest average BTS value (144.51 gf) and LQ04 had the lowest average BTS value (47.17 gf) during seed development. SS showed a significant correlation with seed length, awn length and 1000-seed weight. SS was caused by degradation of abscission layers that formed at early heading stage, and degradation of abscission layers occurred at 14 days after heading. Histological analysis of abscission zone (AZ) showed a smooth fracture surface on the rachilla in high SS genotype, suggesting higher degradation degree of abscission layers. This may resulted from the increased cellulase and polygalacturonase activity found in AZ at seed physiological maturity. Staining of pedicels of two contrasting genotypes suggested more lignin deposition in low SS genotype may play a role in resistance of SS. Furthermore, candidate genes that involved in cell wall-degrading enzyme and lignin biosynthesis were differentially expressed in AZ, indicating the involvement and role in SS. This study provided novel insights into the mechanism of SS in *E. sibiricus.*

## Introduction

Seed shattering (SS) is an important adaptive trait for the efficient propagation of offspring in wild plants, but is a major cause of yield loss in crops (Dong and Wang, 2015). Therefore, during early domestication of grass species (e.g., rice, wheat and barley), loss of SS is considered to be one of the most important traits (Fuller, 2007). In comparison, SS habit of many forage grasses have received little attention from forage breeders, despite SS is a commonly observed trait in many cultivated forage varieties and wild grass species (You et al., 2011; Zhao et al., 2015).

*Elymus sibiricus* L (siberian wildrye) is a perennial, cool-season, self-pollinating, and allotetraploid grass (Dewey, 1974). As an economically important species, *E. sibiricus* has been widely grown and used for pasture and hay, owing to its excellent stress tolerance, good forage quality and adaptability to local environment (Xie et al., 2015). Because of serious SS, however, *E. sibiricus* is difficult to grow for commercial seed production. Previous study showed shattering can cause up to 80% yield losses if harvesting is delayed (You et al., 2011). The provinces of Qinghai and Sichuan produce over 90% (2,400,000 kg) of total *E. sibiricus* seed each year in China. But the average seed production is only 690 kg⋅ha^-1^ due to SS (China Grass Internet, 2013). Selection for seed retention and improvement of SS in this species are, therefore, strongly recommended.

Seed shattering is a highly co-ordinated event involving multiple changes in cell structure, metabolism and gene expression. In many cereals, SS is generally caused by degradation of abscission layers formed at the basal part of grains (Elgersma et al., 1988), and seed retention results from loss of the abscission layers (Thurber et al., 2011). Meanwhile, abscission is associated with cleavage of cell wall components by hydrolases such as CE and PG (Bunya-atichart et al., 2011). CE is the first enzyme responsible for wall loosening at the site of abscission (Roberts et al., 2002), and its activity is associated with organs (e.g., seed, flower, and fruit) abscission. There is a correlation between abscission-specific PG activity and cell separation in plant organs (Huber, 1983) because PG can break down the pectin rich middle lamellae and lead to separation (Taylor and Whitelaw, 2001). To date, several major QTLs and genes for SS have been identified and cloned in cereals. In rice, major shattering genes were reported such as *SH4* (Li et al., 2006), *qSH1* (Konishi et al., 2006), *OsCPL1* (Ji et al., 2010) and *SHAT1* (Zhou et al., 2012). Wheat Q gene regulates plant architecture and seed dispersal (Simons et al., 2006) and overexpression of *TaqSH1* gene regulates floral organ abscission in *Arabidopsis* (Zhang et al., 2013). In maize *Sh1*-orthologous gene was identified as one of the major shattering QTLs (Lin et al., 2012).

In comparison, the mechanism of SS in many forage grasses remains largely unexplored. Limited information is available regarding the formation, development and degradation of the abscission layers, and the histological and physiochemical mechanism, and candidate genes involved in SS remains poorly understood in *E. sibiricus*. To better understand the underlying mechanism of SS, we conducted a combination of morphological and genetic diversity, histological characteristics and hydrolytic enzyme activity as well as candidate genes expression analysis on *E. sibiricus* accessions including cultivars and wild accessions. The results of this study will lead to a better understanding of SS and would be helpful for breeding improvement programs in seed retention for this species.

## Materials and Methods

### Plant Materials

A total of 15 *E. sibiricus* accessions were used in this study. Most of them were from the species range in China, comprising cultivars and wild accessions (**Table 1**). Six plant introduction (PI) accessions were originally obtained from the U.S. Department of Agriculture Germplasm Resources Information Network (GRIN). Other accessions were obtained from the State Key laboratory of Grassland Agro-ecosystems, Sichuan Agricultural University, and Sichuan Academy of Grassland Science. The seeds of each accession were germinated in plastic boxes with moistened blotter paper at room temperature. After germination, seedlings were maintained in a greenhouse under a 25/15°C day/night temperature regimes until they were 8 weeks old. Ten individual plants of each accession were transplanted to the experimental field in Yuzhong campus of Lanzhou University, Gansu, China (latitude 35°34′ N, longitude 103°34′ E, elevation 1720 m). After transplanting plants were well watered immediately and no fertilizer was applied to the plants afterward. To avoid potential seed contaminants, all accessions used in this study were previously identified based on some important phenotypical characteristics such as inflorescence, stem, leaf, and seed. Particularly, this species is small-anthered and long-awned bunchgrass.

**Table 1:** Fifteen *Elymus sibiricus* accessions used in this study.

Code	Accessions	Status	Origin	Morphological characteristics
1	PI504462	Cultivated	Qinghai, China	Low seed shattering, late flowering
2	PI655199	Cultivated	Xizang, China	Low seed shattering, early flowering
3	PI598780	Wild	Kazakhstan	Late flowering
4	Tongde	Cultivar	Tongde, Qinghai, China	Low seed shattering, late flowering
5	Qingmu No.1	Cultivar	Batan, Qinghai, China	Tall, medium seed shattering, late flowering
6	PI499468	Cultivated	Xinjiang, China	Medium seed shattering
7	Chuancao No.2	Cultivar	Hongyuan, Sichuan, China	Tall, early flowering, medium seed shattering
8	Hongyuan	Cultivar	Hongyuan, Sichuan, China	Medium seed shattering, early flowering
9	PI499456	Cultivated	Inner Mongolia, China	Medium seed shattering
10	Y1005	Wild	Ruo ergai, Sichuan, China	High seed shattering
11	PI499453	Cultivated	Inner Mongolia, China	Low seed shattering
12	HZ01	Wild	Hezuo, Gansu, China	Medium seed shattering
13	LQ01	Wild	Luqu, Gansu, China	Medium seed shattering
14	LQ04	Wild	Luqu, Gansu, China	High seed shattering
15	ZhN06	Wild	Zhuoni, Gansu, China	Low seed shattering

### Seed Shattering and Other Seed Related Traits Measurements

The SS of 15 accessions were determined by measuring BTS that required to detach seeds from pedicels (Li et al., 2006), which is inversely proportional to SS degree. Thirty randomly chosen florets from five inflorescences of each plant were examined at each of the five developmental stages, 0, 7, 14, 21, and 28 days after heading (DAH), and their average BTS values were calculated. Other four seed related traits, including SL, SW, AL, KW were measured according to the methods described by Zhao et al. (2015). The mean value, standard deviation (SD), coefficient of variation (CV) of each trait, and correlation analysis among different traits were carried out using Statistical Package for Social Sciences program (SPSS 19.0, SPSS Inc., USA) software. According to the mean value of each trait for each accession, a heatmap was constructed using the Heatmap Illustrator (HemI 1.0) program (Deng et al., 2014). All phenotypic data were normalized under the logarithmic relations at first, due to the different conditions of measurement, then used to map the visualize color matrix. Each line and column represented different accessions and different traits, respectively. Hierarchical clustering was conducted based on average linkage method and the similarity metric with Pearson distance (Deng et al., 2014).

### Histological Analysis of Pedicel Structure

Histological analysis of flower-pedicel structure was carried out at the same five development stages concurrent with SS measurements. In order to reduce variation due to the spikelet position at each developmental stage, the three central spikelets of each florescence were used. The pedicels of each accession were fixed in FAA solution [ethylalcohol (70%): glacial acetic acid: formaldehyde (37∼40%) = 90 : 5 : 5)] (Glime, 2013; Kantar et al., 2016) after vacuum dried for 10 min in a vacuum drier and stored at 4°C in 15 M ethanol. They were then dehydrated in a gradient of ethanol solutions (50, 70, 90, and 100%) for 60 min (Htun et al., 2014). After transparency in the dimethylbenzene and soaking in the paraffin, paraffin-embedded pedicels were sectioned longitudinally to a thickness of 8 μm by the paraffin slicing machine (Jinhua YIDI Medical Appliance CO., LTD, Zhejiang, China). The sections were stained for 20 min with safranin, then 2 min with fast Green (Zhongtai, Shanghai, China). After staining, the pedicel structures were examined using a fluorescence microscope (Olympus Corporation, Japan). In order to detect the relationship between abscission layer development and SS degree at each of five developmental stages, the pedicel junctions after detachments of seed were examined by scanning electron microscopy (Konishi et al., 2006).

### Genetic Diversity of Accessions with Seed Shattering Variations

A total of 15 accessions were sampled and used for genetic diversity analysis using 20 EST-SSR primers. Genomic DNA was extracted from the young leaves according to an SDS method (Shan et al., 2011). The quantity and quality of DNA samples were detected using NanoDrop ND1000 spectrophotometer (Thermo Scientific, USA) and agarose gel electrophoresis, then diluted to 25 ng/μL and stored at -20°. The PCR amplification, SSR genotyping as well as data analysis were carried out using the methods described by Xie et al. (2015). Structure analysis of the 15 accessions was performed using the methods described by Zhang et al. (2016).

### Cell Wall Hydrolytic Enzymes Analysis of the Abscission Zone

The AZ tissues were harvested according to methods described by Li et al. (2006). Each collected flower-pedicel structure consisted of an approximately 1 mm region of the pedicel and 1.5 mm of the flower. The CE and PG were extracted according to the methods described by Awad and Lewis (1980) and Amid et al. (2016). The enzyme activity was assayed in AZs at the same five developmental stages used for BTS and histological analyses, following the manuscript’s protocol of plant CE ELISA kit and plant PG ELISA kit (Shanghai Enzyme-linked Biotechnology Co., Ltd., China), respectively.

### Candidate Genes Expression Analysis

Abscission zone tissues of the two accessions (PI655199 and LQ04) with contrasting SS degree were collected at two of the five developmental stages: 21 days and 28 DAH. The two stages were selected based on results of SS, histological as well as enzyme activity analysis. Each collected flower-pedicel structure consisted of an approximately 1- mm region of the pedicel and 1.5 mm of the flower, which included the AZ (Li et al., 2006). Approximately 30 mg of this AZ tissue was collected. Total RNA was extracted using Plant total RNA Kit (TIANGEN, Beijing, China) according to the manufacturer’s instructions. RNA concentration and quality was measured using an Agilent 2100 Bioanalyzer (Agilent Technologies, Inc., Waldbronn, Germany). Based on the our previous transcriptome data of AZ in *E. sibiricus* (data not published), five candidate genes: *PAL*, *GLU*, *CesA*, *PG* and *XIP1* involved in SS were selected for the gene expression analysis. Gene-specific primers were designed using Primer Express software (Applied Biosystems) and were shown in Supplementary Table S1. The SYBR Premix Ex Taq^TM^ II quantitative PCR system (Takara, Dalian) was applied for qRT-PCR analysis, following the manufacturer’s instructions, and reactions occurred on a Bio-Rad iQ5 real-time PCR instrument (Bio-Rad, Hercules, CA, USA). Expression levels of these candidates were calculated relative to reference gene *GAPDH* using the 2^-ΔΔCt^ method (Livak and Schmittgen, 2001).

## Results

### Morphological and Genetic Diversity of 15 *E. sibiricus* Accessions

**FIGURE 1:**
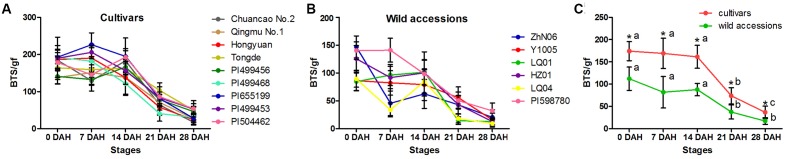
**The breaking tensile strength (BTS) of seeds from the pedicel of *E. sibiricus* accessions at 0, 7, 14, 21, 28 days after heading (DAH): Time-course changes in seed shattering degrees of *E. sibiricus* cultivars (A)** and wild accessions **(B)**, and the average BTS value of cultivars and wild accessions at five developmental stages **(C)**. Bars indicate the mean values ± standard deviation. ^∗^Represent significant difference of BTS value at *p* < 0.01 level.

**FIGURE 2:**
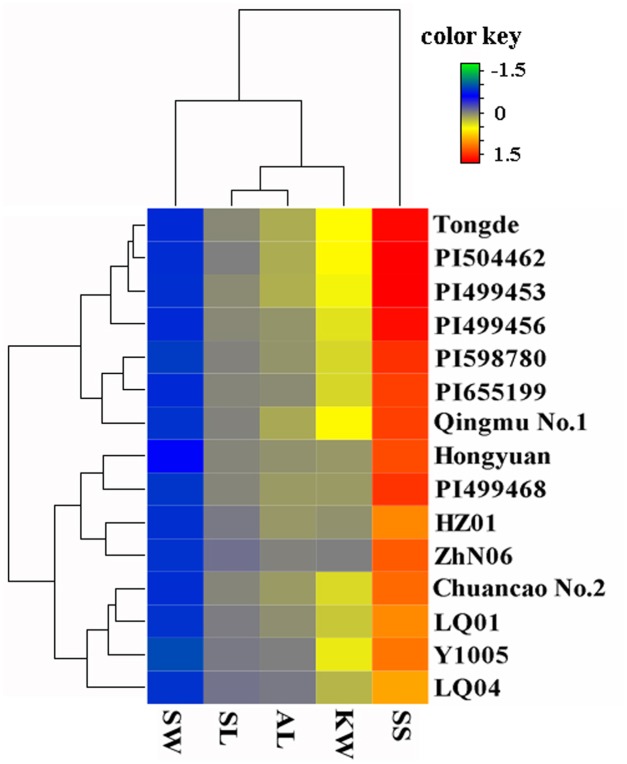
**Clustering and Heatmap analysis of the 15 *E. sibiricus* accessions using morphological traits**. Accessions name are shown in right side of the heatmap. The color scale of heatmap ranged from –1.5 to 1.5 (green to red). Each line and column represents different accessions and different traits, respectively.

The morphological variation and genetic diversity of 15 *E. sibiricus* accessions were studied using five seed related traits: SS, SL, SW, AL and KW, and 20 EST-SSR markers, respectively. Three of five traits: SS, SW and KW had the coefficient variation (CV) value more than 30%. The greatest morphological variation was found for SS (CV = 64.55%), followed by SW (CV = 45.89%), KW (CV = 40.85%), AL (CV = 21.76%) and SL (CV = 14.19%) (**Table 2**). SS showed significant correlation with SL, AL and KW (**Table 3**). The time course changes in SS degree of 15 *E. sibiricus* accessions indicated that BTS value differed at each developmental stage (**Figures 1A–C**). In general, although there was a decline of BTS at 7 DAH for wild accessions, BTS value of cultivars maintained generally a stable level from 0 DAH to 14 DAH. Notably, the average BTS value of wild accessions decreased quickly at 21 DAH, and dropped below 40 gf, while the average BTS value of cultivars remained above 75 gf. At 28 DAH, the BTS value of cultivars varied from 17.51 gf to 53.85 gf, with a mean of 36.78 gf. The BTS value of wild accessions ranged from 9.48 gf to 32.33 gf, with an average of 17.37 gf. Generally, cultivars had higher BTS value than wild accessions, of which PI655199 had the highest average BTS value (144.51 gf) and LQ04 had the lowest average BTS value (47.17 gf) across the whole developmental stages. Clustering analysis based on seed traits can divided most cultivars and wild accessions into two different groups (**Figure 2**). The dendrogram was concordant with morphological variability.

**Table 2:** Morphological variation of five seed related traits among 15 *E. sibiricus* accessions.

Traits	Min	Max	Mean	*SD*	CV (%)
SL (cm)	0.13	1.31	1.01	0.15	14.43
SW (cm)	0.09	1.04	0.16	0.08	47.06
AL (cm)	0.65	2.22	1.27	0.30	23.96
KW (g)	0.96	4.20	2.48	1.02	41.05
SS (gf)	0.00	90.50	29.01	18.73	64.55

*SL, seed length; SW, seed width; AL, awn length; KW, 1000-seed weight and SS, seed shattering; SD, standard deviation; CV, coefficient of variation*.

**Table 3:** The correlation analysis between seed shattering and other seed traits.

Traits	SL	SW	AL	KW	SS
SL	1.000				
SW	0.135	1.000			
AL	0.431^∗∗^	0.064	1.000		
KW	0.047	-0.131	0.552^∗∗^	1.000	
SS	0.240^∗∗^	-0.003	0.414^∗∗^	0.503^∗∗^	1.000

*SL, seed length; SW, seed width; AL, awn length; KW, 1000-seed weight and SS, seed shattering, ^∗∗^means significant correlation*.

**FIGURE 3:**
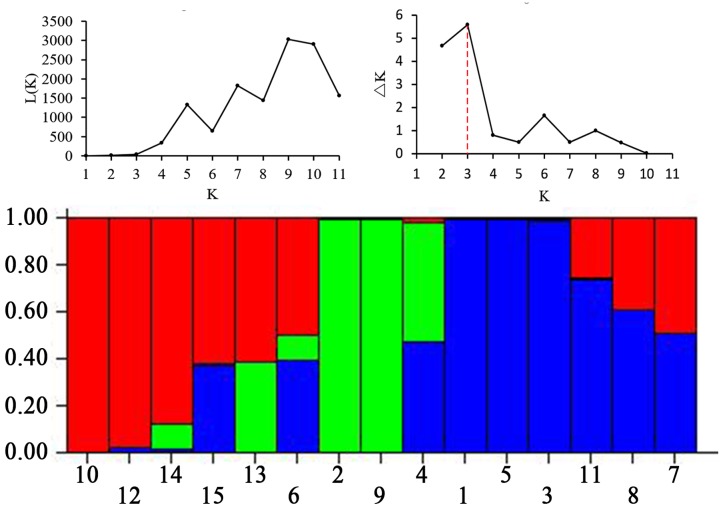
**Three groups of 15 *E. sibiricus* accessions inferred from STRUCTURE analysis and the description of detected the optimum value of *K* by using graphical method**. Three types of color represent different groups. Accessions designations refer to **Table 1**.

The high level of morphological variation was similar to genetic diversity detected by EST-SSR markers. A total of 20 EST-SSR primers amplified 249 bands, with an average of 12.45 per primer. The percentage of polymorphic bands (PPB) was 94.78%, suggesting a high degree of genetic diversity among these accessions (**Table 4**). The value of genetic similarity among accessions ranged from 0.51 (between PI655199 and Y1005) to 0.90 (between “Chuancao No.2” and “Hongyuan”), with an average of 0.72 (**Table 5**). Structure analysis showed all accessions were assigned into three major groups (**Figure 3**). In general, those data suggested rich morphological and genetic variation, and provided valuable germplasm resources from which the mechanisms of SS may be investigated.

**Table 4:** Genetic diversity analysis of 15 *E. sibiricus* with seed shattering variation by EST-SSR markers.

Primer ID	Forward primer (5′-3′)	Reverse primer (5′-3′)	TB	MB	PB	PPB (%)
Elw1420s081	GGATAGACCCATGAGCTGACTGAT	CTTTCTCCACAAGTTGAACACAACA	11	0	11	100.00
Elw1468s087	TAGCAATAAGTTGCTGCTGCTGTT	CCACCTCTAAATTAATCACCACGAA	12	0	12	100.00
Elw2594s136	AAATGTCAACGACGAAAAAGGAAA	ATGTAGCCTTGAGAACACTGGTCC	10	0	10	100.00
Elw3384s187	AGCTCCTGATAGAAAGAGCCATCA	GGCTGCTGGAACTGAAGACAGTA	12	0	12	100.00
Elw3545s194	CAGCACTAGTATCCACCTCCACCT	TGTTACAGCCTCTTCAGGCTCTTC	5	2	3	60.00
Elw3592s195	TGTTGACAAAAGCAGTTGAAGGG	GATTTGACCATGGACTGCTTCAC	13	1	12	92.31
Elw3995s226	CTCTAGGGTTTTGGGATTTTAGCC	GTTGTGGAGGTCGGAGAAGGT	9	1	8	88.89
Elw4021s228	TTCAAACCACAAGAGGAGAAGGAC	TGGTGGTGGTAGTATTGGTTGTTG	8	1	7	87.50
Elw5447s306	TCCTCAAACTCCTCCTCTCTTCG	GAGGTAAGTCTCGACATCCTCGAC	27	0	27	100.00
Elw5627s404	AGATGAAGCTGGTAACCGAGACAG	ATTTCCTCTAATGGAAGCTCTGGC	23	0	23	100.00
Ps1475	CCCCAGTCTCCTCCATACATACAC	GTCTTGCCCGGAAAATTTACCTAC	10	1	9	90.00
Ps1830	GACTCGGCGAAAGGACTCTCT	CTCGACGTCCTTCATGAGCTT	19	2	17	89.47
Ps2117	TCCAAGACCTCGGTACTGGAAC	CTGATGTAGGTACGGTCCTGCTCT	7	0	7	100.00
Ps2283	GCCACAACAAGAGAAGACCTTGC	GACCTGCATGATGCTCTCGC	29	0	29	100.00
Ps261	CTCGAATCCAGCTGAACAATTTCT	AGTCGATCCTCACCTTCATCTCC	10	0	10	100.00
Ps3447	AGCTTTATGAAGATCGCCACTCAC	CTGCTGCTGCTACCGTTCTTATTT	18	0	18	100.00
Ps3577	CATCTTGCATATAGCTCCTTCGCT	CTCAAGAAACCCACAATCCAATTC	6	1	5	83.33
Ps938	TTGCTCCTATGGTTCCACGTAGTT	AAAGTGAAATTCTGCCATCAGAGC	9	0	9	100.00
Ltc0055	AAGAAGAAGAGGCCGAGGAATAAA	CGTGGATGTGCTGCAGGTAGTA	4	3	1	25.00
Ltc0157	GCAATGAACACTGAATCAATCGAG	CGTGTGAGACTCATCGATGTTACC	7	1	6	85.71
Mean			12.45	0.65	11.80	90.11
Total			249	13	236	94.78

*TB, MB, PB and PPB represented respectively TB, total number of amplified band; MB, number of monomorphic bands; PB, number of polymorphic bands and PPB, the percentage of polymorphic bands*.

**Table 5:** Nei’s original measures of genetic identity and genetic distance.

ID	1	2	3	4	5	6	7	8	9	10	11	12	13	14	15
1		0.56	0.88	0.73	0.89	0.78	0.81	0.82	0.63	0.73	0.83	0.76	0.65	0.72	0.81
2	0.58		0.64	0.53	0.59	0.58	0.53	0.57	0.66	0.51	0.58	0.52	0.57	0.54	0.56
3	0.13	0.44		0.71	0.89	0.77	0.78	0.80	0.66	0.74	0.84	0.76	0.67	0.74	0.82
4	0.32	0.64	0.35		0.79	0.70	0.69	0.68	0.65	0.66	0.71	0.64	0.66	0.67	0.69
5	0.11	0.53	0.12	0.24		0.79	0.81	0.82	0.66	0.74	0.90	0.78	0.67	0.73	0.82
6	0.25	0.54	0.26	0.36	0.24		0.76	0.78	0.61	0.70	0.78	0.76	0.67	0.73	0.80
7	0.21	0.64	0.25	0.37	0.21	0.27		0.90	0.60	0.78	0.78	0.76	0.66	0.74	0.78
8	0.20	0.57	0.22	0.39	0.20	0.25	0.10		0.61	0.76	0.80	0.75	0.63	0.72	0.78
9	0.46	0.41	0.42	0.43	0.42	0.50	0.51	0.50		0.62	0.67	0.60	0.64	0.60	0.62
10	0.32	0.67	0.30	0.42	0.30	0.35	0.25	0.27	0.47		0.75	0.80	0.70	0.73	0.78
11	0.18	0.55	0.17	0.34	0.11	0.25	0.25	0.22	0.40	0.29		0.82	0.69	0.74	0.82
12	0.27	0.66	0.27	0.44	0.24	0.28	0.27	0.29	0.50	0.22	0.20		0.71	0.75	0.84
13	0.43	0.57	0.40	0.42	0.40	0.40	0.42	0.46	0.45	0.36	0.37	0.35		0.80	0.73
14	0.33	0.61	0.30	0.40	0.31	0.32	0.30	0.33	0.51	0.32	0.31	0.29	0.22		0.76
15	0.21	0.58	0.20	0.37	0.20	0.22	0.25	0.25	0.47	0.24	0.19	0.18	0.32	0.27	

*Nei’s genetic identity (above diagonal) and genetic distance (below diagonal). Accessions designations refer to **Table 1***.

### Histological Analysis of Pedicel Structure

**FIGURE 4:**
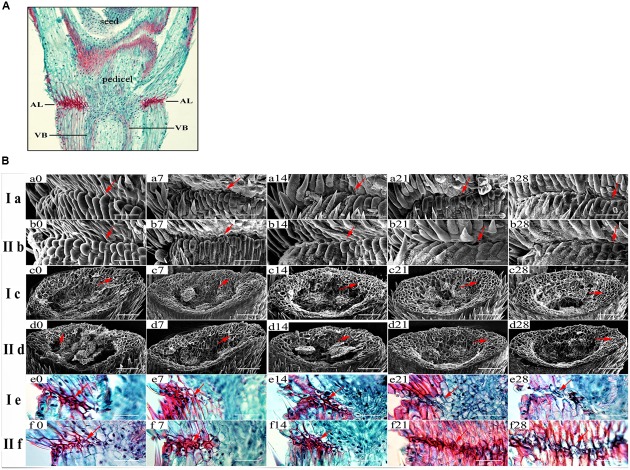
**Histological analysis of abscission zone: Pedicel histological structure and abscission layer position in pedicel **(A)**. (B)** Ia and IIb show scanning electron photos of boundary regions between pedicel and seed. Ic and IId show scanning electron photos of pedicel junction after detachment of seeds in high seed shattering genotype LQ04 and low seed shattering genotype PI655199 at five developmental stages. Ie and IIf show longitudinal sections across the abscission zone of high seed shattering genotype and low seed shattering genotype, respectively. Sections were stained with safranin-fast green, and lignin in dark red. Degradation of abscission layer occurs at 21 and 28 days after heading (e21, e28). Bar is 50 μm in Ia, IIb, Ie and IIf and 100 μm in Ic, IId. Red arrow indicates abscission layer location.

In order to further detect the relationship between SS and degradation degree of abscission layer, we conducted the histological analysis of pedicel structure of 15 *E. sibiricus* accessions with SS variation. Anatomical investigation with longitudinal sections indicated abscission layers were already present at heading in all 15 *E. sibiricus* accessions. They occurred on both sides of vascular bundle, which could be stained dark red by safranin (**Figure 4A**). The shape and arrangement orientation of abscission layers were different when compared with surrounding cells. Generally, abscission layers had two or three layers of cells that had an elliptic shape and a well-organized shape. In addition, the cell size of abscission layer was smaller than that of the parenchyma cells in the rachilla. For most accessions, degradation of abscission layers occurred at 14 DAH. LQ04 had more serious degradation of abscission layers than low SS genotype PI655199 at 28 DAH. A well-defined boundary between pedicel and flower was present in high SS genotype at 21 and 28 DAH (**Figure 4B**, Ia, IIb). Additionally, scanning electron microscopy showed a smooth fracture surface on the rachilla in high SS genotype at 21 and 28 DAH while rough and irregular surface was observed in low SS genotype (**Figure 4B**, Ic, IId). Based on these staining results, lignin content increased along with five developmental stages, there was more lignin in the AZ and surrounding pedicel tissue of low SS genotype PI655199 than in high SS genotype LQ04 at 21 and 28 DAH (**Figure 4B**, Ie, IIf).

### Cell Wall Hydrolytic Enzymes Analysis of Abscission Zone in 15 *E. sibiricus* Accessions

**FIGURE 5:**
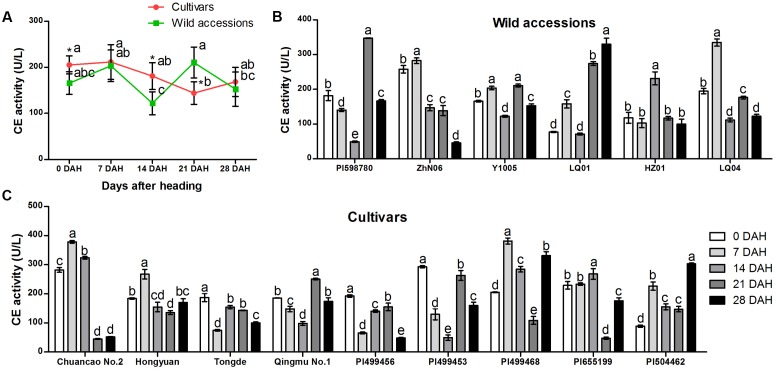
**Specific activity of cellulase in abscission zone of different *E. sibiricus* accessions at 0, 7, 14, 21, and 28 days after heading (DHA)**. Average CE activity of cultivars and wild accessions at five developmental stages **(A)**. Time-course changes in CE activity of wild accessions **(B)** cultivars **(C)**. Bars indicate the mean values ± standard deviation. ^∗^Represents significant difference of CE activity value at *p* < 0.01 level.

Specific activity of CE varied among 15 *E. sibiricus* accessions at different stages during seed development (**Figures 5A–C**). Generally, CE exhibited a different trend of activity between cultivars and wild accessions. The biggest differences of CE activity between cultivars and wild accessions were observed at 14 and 21 DAH. For cultivars, the mean CE activity was higher (181 U/L) than wild accessions (122 U/L) at 14 DAH while that activity was lower (144 U/L) than wild accessions (210 U/L) at 21 DAH (**Figure 5A**).

During the first 7 days after heading, the CE activity value was similar between cultivars and wild accessions and were maintained at more than 165 U/L (**Figure 5A**). The CE value of cultivars and wild accessions began to decrease after 7 DAH. The CE value of wild accessions began to increase rapidly at 14 DAH, and reached the highest value (210 U/L) at 21 DAH and remained above 150 U/L at 28 DAH. In comparison, the CE value of cultivars decreased quickly at 7 DAH, and dropped below 150 U/L at 21 DAH. At the same stage, wild accessions had the highest CE value.

**FIGURE 6:**
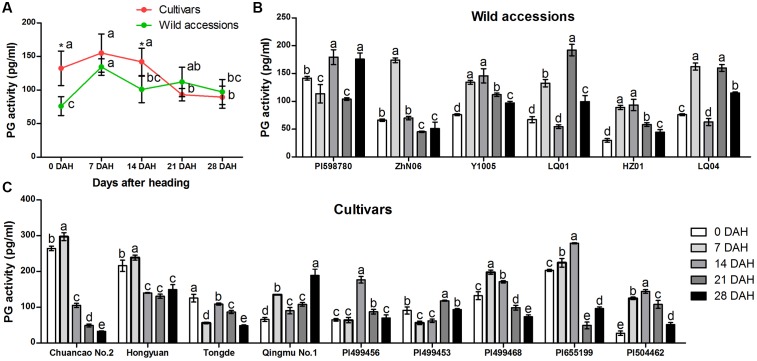
**Specific activity of polygalacturonase (PG) in abscission zone of different *E. sibiricus* accessions at 0, 7, 14, 21, and 28 days after heading**. Average PG activity of cultivars and wild accessions at five developmental stages **(A)**. Time-course changes in PG activity of wild accessions **(B)** and cultivars **(C)**. Bars indicate the mean values ± standard deviation. ^∗^Represents significant difference of PG activity value at *p* < 0.01 level.

Similarly, specific activity of PG varied among 15 *E. sibiricus* accessions at different stages during seed development (**Figures 6A–C**). The biggest differences of PG activity between cultivars and wild accessions were found at 0 and 14 DAH. For the cultivars, the mean PG activity was higher (132 pg/mL and 142 pg/mL) than wild accessions (76 pg/mL and 101 pg/mL) at 0 and 14 DAH. But, at 21 and 28 days after heading, the average PG activity of wild accessions was higher than cultivars, and their value were between 97 pg/mL and 112 pg/mL (**Figure 6B**).

**FIGURE 7:**
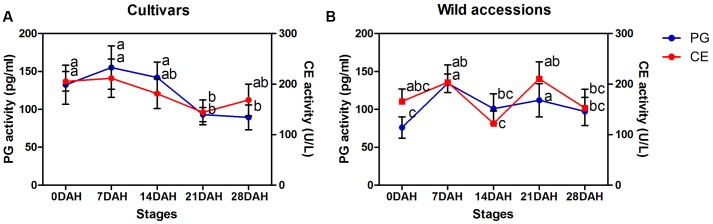
**Time-course changes in celluase and polygalacturonase activity at each of five developmental stages (0, 7, 14, 21, and 28 days after heading) in cultivars (A)** and wild accessions **(B)**. Bars indicate the mean values ± standard deviation.

Generally, PG and CE activity varied at each of five developmental stages for both cultivars and wild accessions, but they exhibited similar trend of activity during seed development (**Figures 7A,B**), indicating their roles and interaction in differentiation of SS. High SS accessions showed higher CE and PG activity at seed maturity stage (21 and 28 DAH). For example, high SS genotype LQ04 (CE = 176 U/L, PG = 159 pg/mL) had higher CE and PG activity than low SS genotype PI655199 (CE = 47 U/L, PG = 49 pg/mL) at 21 DAH.

### Candidate Genes Expression in Abscission Zone

**FIGURE 8:**
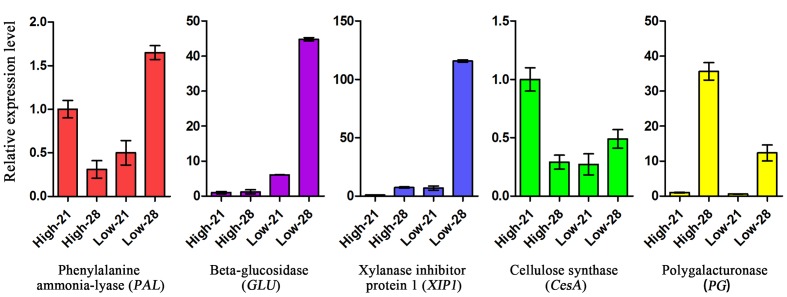
**Candidate genes expressions in abscission zone of two *E. sibiricus* genotypes with contrasting seed shattering**. Bars indicate the mean values ± standard deviation.

Five SS candidate genes including *PAL*, *GLU*, *XIP1* and CesA and *PG* were selected for expression analysis. The results showed that all these genes were differentially expressed in the AZ at two development stages, 21 day and 28 DAH (**Figure 8**). We used High-21 as a benchmark for relative expression analysis. *CesA* were down-regulated in low SS genotype PI655199 at 28 DAH (Low-28). The expression of other four genes (*PAL*, *GLU*, *XIP1* and *PG*) was up-regulated in Low-28. The relative expression of *PG* for high SS genotype LQ04 was higher than that of low SS genotype PI655199 at 28 DAH. In particular, the relative expression of *XIP1* for PI655199 at 28DAH was almost 116 times higher than that of LQ04 at 21 DAH (Low-21). These results indicated the involvement and role of these candidate genes in SS.

## Discussion

### Correlation between Seed Shattering and Other Seed-related Traits

Seed shattering is a commonly observed trait in wild grass species and many forage cultivars (Zhao et al., 2015). This study showed wide variation in SS among 15 accessions. The BTS value decreased quickly after 14 DAH, and reached the lowest BTS value at 28 DAH. Cultivars had higher average BTS value than wild accessions during seed development. Various direct and indirect factors influencing SS were reported, i.e., the size, strength, shape, and the flexibility of the glumes and compactness of the inflorescence and spikelets (McWilliam, 1980). In this study, we found a significant correlation between SS and three seed related traits: AL and KW and SL, similar to previous reports. The awn is a morphological characteristic found in most cereal crops, such as rice, wheat, barley, and sorghum. In wild plants, long awns have been reported to aid in seed dispersal, however, long awn is disadvantageous for grain harvest (Magwa et al., 2016). In practice, high yielding plants are likely more susceptible to shattering, if harvest is delayed (Elgersma et al., 1988). Many studies also indicated the expression of SS may be modified by environmental factors such as a deficit or surplus of water, high/low temperature (Taylor and Whitelaw, 2001; Taghizadeh et al., 2009). Consequently, in practice wide germplasm collections, recurrent selection with multiple location and year testing, large seed field trials should be important to selection for seed retention and improvement of SS in this species.

### Histological and Hydrolytic Enzymes Activity Difference of Abscission Zone

Previous studies have showed that abscission was generally caused by the development of abscission layer, and revealed a correlation between the SS and the degree of degradation for abscission layers (Jin et al., 1982; Ebata and Tashiro, 1990; Jin et al., 1992; Fukuta et al., 1994a,b; Oba et al., 1995; Watanabe et al., 2003; Konishi et al., 2006; Li et al., 2006; Akasaka et al., 2011). In rice, abscission layers were described as a band of small cells that begin to form before heading and finish cell expansion to maximum size at the stage of heading (Jin, 1986; Oba et al., 1995; Roberts et al., 2002; Watanabe et al., 2003; Lin et al., 2007). Based on our results abscission layers were already present at heading in *E. sibiricus*, and included two small cell layers, which were considered to be more advantageous to cell separation and breakage formation (Gubert et al., 2014). For all accessions tested, seed didn’t shed at heading stage but began to shed at 21 DAH. Histological analysis of AZ at 21 and 28 DAH showed different degradation degree among different accessions. High SS accessions had a smooth fracture surface of rachilla when compared with low SS genotypes, suggesting the high level of degradation. This may resulted from the increased hydrolytic enzymes activity found in AZ. Abscission is related to cleavage and degradation of cell wall components by two major cell wall hydrolytic enzymes PG and CE (Fuller et al., 2010; Liljegren, 2012; Estornell et al., 2013; Tada et al., 2015). PG and CE have a crucial effect on the degradation of abscission layer, which can contribute to the shattering of seed and other organs (Sexton et al., 1980; Kalaitzis et al., 1997; Agrawal et al., 2002). PG have been implicated as positive regulators of cell separation, fruit ripening, abscission, cell growth and dehiscence (Jenkins et al., 1999; Sander et al., 2001; Atkinson et al., 2002, 2012; González-Carranza et al., 2007; Xiao et al., 2014). Typically, a research reported that decreasing PG activity could reduce seed loss prior to harvest (Swain et al., 2011).

In our study, PG and CE activity varied across five developmental stages. Similar trend of activity was found in pod shattering process of soybean (Agrawal et al., 2002). Wild accessions had higher PG and CE activity when compared with cultivars at seed physiological maturity, indicating the involvement and roles of PG and CE in SS difference among cultivars and wild accessions. A balance and interaction between PG, CE and other enzymes may be the key factor that regulates and determines the abscission process. Previous studies showed PG can hydrolyse specially pectin which is a dominant component and a fiber-enforced composite material in plant cell wall and has important consequences for cell adhesion (Chanliaud and Gidley, 1999; Wen et al., 1999; Willats et al., 2001; Daher and Braybrook, 2015). Then the decreased PH resulted from cell wall pectin hydrolyzation, in turn, might pose several direct potentially and indirect consequences for wall properties and other enzyme activities (Willats et al., 2001; Pelloux et al., 2007; Wolf and Greiner, 2012). Thus, the consequences might elevate CE activity and give rise to a stronger hydrolyzation and degradation of abscission layer cell wall. Our results indicated that CE and PG exhibited similar trend of activity during seed development, which could confirm partially an interaction between PG and CE in hydrolyzing cell wall.

### Lignin Deposition and Seed Shattering

Lignin is a highly branched polymer of phenylpropanoid compounds, and a component of the plant cell wall. The secondary cell wall provides the strength necessary to make cells suitable for transport and support which is imparted by the presence of lignins in the secondary cell walls (Barceló, 1997; Rogers and Campbell, 2004). In grass species, lignin comprises approximately 20% of the secondary cell wall, filling pores between the polysaccharides (Vogel, 2008). Lignin plays an important roles in plant growth, development and defense responses. Lignin deposition generally occurs when cell growth is completed and the cell wall undergoes secondary thickening (Rogers and Campbell, 2004; Wang et al., 2013). Plants are exposed to different stress, which may change lignin content and composition (Moura et al., 2010). It has been believed that lignification is a mechanism for stress defense in plants, particularly for plant–microbe interactions, this has been suggested as defense responses of host plants to the stress (Bhuiyan et al., 2009). Lignin is also identified as a major factor in recalcitrance of cell walls to digestion, particularly during enzymatic hydrolysis (Chen and Dixon, 2007). A previous study in rice also showed that SS can be induced by inhibiting lignin biosynthesis, and decreasing lignin levels in the AZ and surrounding pedicel tissues could lead to an increase in SS (Yoon et al., 2014). At seed physiological maturity, the total seed weight pressing the underlying abscission layers increases, which may result in easier breaking of abscission layers with lower lignin content. In this study, staining of pedicels of two contrasting genotypes at 21 and 28 days after heading showed that lignin deposition was much lower in high SS accession than in low SS accession. Meanwhile, high SS accession had lower BTS value when compared with low SS accession. These results implied higher lignin deposition found in low SS genotype may play a role in resistance to SS.

### Candidate Genes Involved in Seed Shattering

The shattering habit is a complex polygenic trait that is controlled by many genes (Konishi et al., 2006). Previous studies showed many glycosyl hydrolase family genes play an important role in modifying plant cell wall structure and component during tissue development (Zhou et al., 2006). CE (1,4,-β-glucanase), the first hydrolytic enzyme reported, play a critical role in wall loosening during abscission (Roberts et al., 2002). In rice, the gene *OsCel9D* (synonym *OsGLU1*) encodes an endo-1,4,-β-glucanase gene with cellulose function, and mutations of this gene reduce cell elongation and cellulose content, and increase the pectin content, therefore hampering the abscission process in SS (Zhou et al., 2006). Xylan is the major component of hemicelluloses. Xylanase inhibitors (*XIs*) can inhibited the activity of xylanase, which catalyze the hydrolysis of the β-1,4-xylosidic bonds in xylan (Xin et al., 2014). The low SS genotype PI655199 showed much higher expression of *XIP1* when compared with high SS genotype. This indicated that the expression of this gene might be associated with a reduction of SS. *PAL* is the key gene required for monolignol biosynthesis, suppression of this gene took place early in the monolignol biosynthetic pathway, which causes significant reduction in lignin content (Chen and Dixon, 2007). A similar result was found in this study, where *PAL* was down-regulated and lignin content was relatively lower in high SS genotype LQ04, corresponding to increased SS. The different expression of these candidate genes indicated the involvement and role in SS, but which genes played a key role in difference of SS in *E. sibiricus* still remains unknown.

## Conclusion

This study investigated the differences in SS of cultivars and wild accessions in relation to morphological and genetic diversity, histological characteristics, lignin staining, cell wall hydrolytic enzymes activity and candidate genes expressions. Wide variation in the tendency for SS was found among *E. sibiricus* cultivars and wild accessions. In general, cultivars had higher average BTS value than wild accessions. SS is caused by a degradation of the abscission layer formed early at heading stage. Histological analysis of AZ showed a smooth fracture surface on the rachilla in high SS genotype due to higher degradation degree of abscission layers. This may resulted from the increased CE and PG activity found in AZ at seed physiological maturity. Meanwhile more lignin deposition found in low SS genotype may play a role in resistance of SS. Furthermore, candidate genes that involved in cell wall-degrading enzymes, and lignin biosynthesis were differentially expressed in AZ, indicating the involvement and role in SS. This study provided novel insights into the mechanism of SS in *E. sibiricus.*

## Availability of Data and Materials

The datasets supporting the conclusions of this article are included within the article and its additional files.

## Author Contributions

WX and YW designed the experiments and provided guidance of the study. XZ, JZ, and ZZ performed the experiments. ZZ and JZ provided assistance in the data analysis. WX and XZ wrote and revised the manuscript. All authors have read and approved the final manuscript.

## Conflict of Interest Statement

The authors declare that the research was conducted in the absence of any commercial or financial relationships that could be construed as a potential conflict of interest.

## Abbreviations

AL: awn length
AZ: abscission zone
BTS: pedicel breaking tensile strength
CE: cellulase
CesA: Cellulose synthase
GLU: beta-glucosidase
KW: 1000-seed weight
PAL: phenylalanine ammonia-lyase
PG: polygalacturonase
qRT-PCR: quantitative real-time PCR
SDS: sodium dodecyl sulfate
SL: seed length
SW: seed width
XIP1: xylanase inhibitor protein 1
